# Glycemic Excursions in Type 1 Diabetes in Pregnancy: A Semiparametric Statistical Approach to Identify Sensitive Time Points during Gestation

**DOI:** 10.1155/2017/2852913

**Published:** 2017-02-08

**Authors:** Resmi Gupta, Jane Khoury, Mekibib Altaye, Lawrence Dolan, Rhonda D. Szczesniak

**Affiliations:** ^1^Division of Biostatistics & Epidemiology, Cincinnati Children's Hospital Medical Center, Cincinnati, OH, USA; ^2^Division of Endocrinology, Cincinnati Children's Hospital Medical Center, Cincinnati, OH, USA; ^3^Department of Pediatrics, University of Cincinnati, Cincinnati, OH, USA

## Abstract

*Aim.* To examine the gestational glycemic profile and identify specific times during pregnancy that variability in glucose levels, measured by change in velocity and acceleration/deceleration of blood glucose fluctuations, is associated with delivery of a large-for-gestational-age (LGA) baby, in women with type 1 diabetes.* Methods.* Retrospective analysis of capillary blood glucose levels measured multiple times daily throughout gestation in women with type 1 diabetes was performed using semiparametric mixed models.* Results.* Velocity and acceleration/deceleration in glucose levels varied across gestation regardless of delivery outcome. Compared to women delivering LGA babies, those delivering babies appropriate for gestational age exhibited significantly smaller rates of change and less variation in glucose levels between 180 days of gestation and birth.* Conclusions.* Use of innovative statistical methods enabled detection of gestational intervals in which blood glucose fluctuation parameters might influence the likelihood of delivering LGA baby in mothers with type 1 diabetes. Understanding dynamics and being able to visualize gestational changes in blood glucose are a potentially useful tool to assist care providers in determining the optimal timing to initiate continuous glucose monitoring.

## 1. Introduction

Pregnancy in women with type 1 diabetes mellitus is associated with an increased risk of various adverse outcomes—both for mothers and for their offspring. These morbidities include preeclampsia, preterm delivery, cesarean section delivery, and large-for-gestational-age (LGA) infant [1, 2]. Long-term adverse health outcomes in the offspring have also been reported such as obesity, insulin resistance, beta cell dysfunction, type 2 diabetes mellitus, and cardiovascular dysfunction [3–5]. Management of pregnant women with diabetes focuses on the importance of good glycemic control before and during pregnancy, to decrease the frequency of adverse outcomes for both infants and mothers [6, 7]. Tracking maternal glycemic control over the entire pregnancy has provided insights into the effects of maternal glucose control on various neonatal outcomes, including LGA babies [8]. LGA babies born to mothers with diabetes are likely to be significantly overweight both in childhood and as adults, putting them at risk of developing type 2 diabetes [9].

In recent years, advancement in technology has improved diabetes self-management practices allowing monitoring of blood glucose on both a programmed and continuous basis. The sheer volume of data produced and the variability that these data contain have proved challenging to analyze and interpret. Regulation of glucose is a dynamic process that varies in response to meals as well as gestational period. The maternal glucose profile may be considered as measurement of a continuous-time monitoring process whose outputs are samples of functions or curves. Each curve alludes to blood glucose oscillations that occur throughout the day and perhaps at the same time on different days throughout pregnancy. In recent studies, glucose variabilities have been attributed to both hyperglycemic spikes and hypoglycemic troughs [10, 11]. Both have been attributed to a wider range of chronic and severe complications associated with diabetes mellitus and hence can provide crucial information about risk factors for the development of complications in diabetes. Different methods, such as calculating the standard deviation (SD), coefficient of variation (COV), and mean amplitude of glycemic excursion (MAGE), are popular approaches to measure and monitor fluctuations or glycemic excursions in blood glucose levels [12]. These approaches, though computationally straightforward, have drawbacks in investigating highly nonlinear profiles [13, 14]. For example, SD is sensitive to outliers and may overestimate the magnitude of a glycemic excursion. Using COV, which is computed based on SD, or using MAGE, which only takes peaks and nadirs at greater than one SD, can also lead to misleading estimation of glycemic excursions.

Vandyke et al. [15] used semiparametric mixed models to assess time-specific differences in the glycemic profiles of mothers with type 1 diabetes who delivered LGA and appropriate-for-gestational-age (AGA) babies. LGA and AGA profiles had greatest distinctions in the first and third trimesters of pregnancy. The study showed that there is a difference in slopes between glycemic profiles arising from LGA and AGA births, suggesting that rate of change (i.e., velocity) and acceleration (or deceleration) of glucose levels vary throughout pregnancy. Even though the above study estimated the mean curvature in the entire glycemic profile, which avoided the typical loss of information with using summary measures, it did not examine the complex form of time and subject-specific rates of change within the glucose profiles.

Analysis of the first- and second-order rates of change is a way to evaluate the dynamics of blood glucose fluctuations on the gestational time scale. First-order rates of change, which are obtained by taking the derivative of estimated glucose trends over pregnancy, correspond to the velocity of glycemic fluctuations. Large rates of change imply rapid blood glucose fluctuations and hence a less stable system. Second-order rates of change indicate how rapidly these fluctuations occur, estimating them in terms of acceleration or deceleration. The acceleration of glucose, which is computed as the derivative of the glucose level, can be described as a change in glucose velocity (rate of change) over time. Acceleration (and deceleration) can be termed as a measure of variability, which indicates the change in curvature of maternal glycemic profiles across gestation. Identifying first- and second-order glucose rates of change with respect to gestational time will be helpful in allowing treatment to be targeted to precise points during pregnancy.

We hypothesize that estimating glycemic variability in terms of derivatives will provide better insight into hypoglycemia and hyperglycemia excursions, which have been shown to be associated with various adverse outcomes and chronic diabetes complications [16]. Measures of oxidative stress, such as reactive oxygen species, which have been associated with hyperglycemia, are closely related to glucose peaks and fluctuating high- and low-glucose concentrations [17].

Being able to estimate rate of change and acceleration in glucose levels across specific gestational periods can provide insight into glycemic fluctuation and risk of hyperglycemia and hypoglycemia at specific gestational time periods on a continuous basis. The purpose of this study is threefold: (1) to develop a semiparametric mixed model [18] to assess the precise timing and degree of rapid fluctuations in the glycemic profiles of mothers with type 1 diabetes; (2) to contrast this novel approach with conventional glucose variability calculations; and (3) to determine the extent to which these specific fluctuations are associated with delivery of LGA baby.

## 2. Methods

### 2.1. Participant Characteristics and Study Variables

The study methods and cohort characteristics have been described in detail elsewhere [6]. Briefly, pregnant women or women who were planning a pregnancy and had type 1 diabetes were recruited and enrolled in the study. The women were prospectively followed as part of a 17-year interdisciplinary program of diabetes in pregnancy between 1978 and 1995 at the University of Cincinnati Medical Center. The study subjects were managed with intensive insulin therapy, involving a split mixed-dose regimen of three to four injections per day using short- and intermediate-acting insulin combined with dietary regulation. After 1981, women were instructed to check blood glucose concentrations 6–8 times a day: while fasting, preprandially (before each meal), 90 min postprandially (after each meal), at bedtime, and occasionally at 3:00 AM. This analysis included women who used a reflectance meter through pregnancy and delivered a singleton live fetus beyond 32 weeks of gestation. The glucose measurements recorded between gestational days 50 and 250 were included in the study. Data from profiles corresponding to neonatal death within 28 days of delivery or presence of a major congenital malformation were excluded from the study. Birthweight was measured within the first hour of delivery using an electronic scale (Toledo Scale, Worthington, Ohio). LGA was defined as birth weight greater than the 90th percentile for gestational age, based on race- and gender-specific growth curves. We estimated the rate of change and rate of acceleration (or deceleration) in mother's glycemic profiles with respect to gestational time for LGA and AGA outcomes separately by taking respective first- and second-order derivatives of the model equations. A semiparametric mixed effects model using penalized regression splines with a cubic truncated power basis was employed to provide smooth estimates of the longitudinal glycemic profiles [19, 20]. Analyses were implemented in SAS 9.4 (SAS Institute, Cary, NC). Implementation is provided in the online supplement (see the appendix, in Supplementary Material available online at https://doi.org/10.1155/2017/2852913). We considered LGA and AGA profiles to have different trends across gestation and characterized them as described below.

### 2.2. Model Setup and Notation

First, we set up a semiparametric mixed model to examine glucose overgestation. The observed glucose recordings from the *i*th pregnancy and *j*th measurement observed at gestation *t*_*ij*_ can be expressed in the semiparametric mixed effects model, separately for LGA and AGA profiles, as

(1) YijLGA=β0+β1tij+β2tij2+β3tij3+β02LGAi+β12LGAitij+β22LGAitij2+β32LGAitij3+∑l=1Lbltij−Kl+3+γ0i+γ1itij+εij,YijAGA=β0+β1tij+β2tij2+β3tij3+∑l=1Lbltij−Kl+3+γ0i+γ1itij+εij.

The term LGA_*i*_ indicates whether the subject delivered LGA or AGA baby (1 = LGA, 0 = AGA). The polynomial terms coefficients, *β*_0_, *β*_1_, *β*_2_, and *β*_3_, are for the respective, intercept, slope, quadratic, and cubic terms to model global curvature of the glycemic profile. The summed expression is comprised of the knots at distinct locations *K*_*l*_  (*l* = 1,…, *L*) evaluated at gestational time *t*_*ij*_ with corresponding coefficients *b*_1_,…, *b*_*l*_. The expression (*t*_*ij*_ − *K*_*l*_)_+_ is the positive part of the function (i.e., max⁡{0, *t*_*ij*_}). To account for variation for both between- and within-subject terms, random intercept (*γ*_0*i*_) and slope (*γ*_1*i*_) terms were included in the model. In accordance with semiparametric model fitting strategy, the smoothing parameters are incorporated as random effects. We assumed that the random effects are mutually independent and distributed as *b*_*l*_ ~ *N*(0, *σ*_*b*_^2^), *γ*_0*i*_ ~ *N*(0, *σ*_*γ*_0__^2^), *γ*_1*i*_ ~ *N*(0, *σ*_*γ*1_^2^), and measurement error *ε*_*ij*_ ~ *N*(0, *σ*_*ε*_^2^). For this analysis we considered the same spline coefficients and the same degree of smoothing for both LGA and AGA profiles (i.e., *b*_*l*_^LGA^ = *b*_*l*_^AGA^ and Var(*b*_*l*_^LGA^) = Var(*b*_*l*_^AGA^)).

Next, we computed the rate of change in glucose levels for women with LGA and AGA births corresponding to the first-order derivative with respect to gestation. More specifically, rate of change, or velocity, can be thought of as change in glucose levels over the whole gestational time period, indicating the speed at which glucose levels are changing throughout pregnancy.

The rate of change in glucose levels across gestation for LGA_*i*_ can be written as the first derivative of the model:

(2) dYijLGAdtij=β1+2β2tij+3β3tij2+β12LGAi+2β22LGAitij+3β32LGAitij2+3∑l=1Lbltij−Kl+2.

Similarly, the rate of change in glucose levels across specific gestational periods for mothers with AGA babies can be expressed as

(3) $$\begin{array}{c} \frac{d\left(Y_{ij}^{\text{AGA}}\right)}{dt_{ij}}=\beta_{1}+2\beta_{2}t_{ij}+3\beta_{3}t_{ij}^{2}+3\overset{L}{\underset{l=1}{\sum }}b_{l}{\left(\;t_{ij}-K_{l}\;\right)}_{+}^{2}. \end{array}$$

The acceleration/deceleration, or the second-order derivative with respect to gestation, was computed to measure how fast (or slow) the velocity of glucose levels is changing across gestation.

And the acceleration in glucose levels across gestational time for LGA_*i*_ is

(4) d2YijLGAdtij2=2β2+6β3tij+2β22LGAi+6β32LGAitij+6∑l=1Lbltij−Kl+.

The acceleration in glucose levels for mothers with AGA births is

(5) $$\begin{array}{c} \frac{d^{2}\left(Y_{ij}^{\text{AGA}}\right)}{d{t_{ij}}^{2}}=2\beta_{2}+6\beta_{3}t_{ij}+6\overset{L}{\underset{l=1}{\sum }}b_{l}{\left(\;t_{ij}-K_{l}\;\right)}_{+}. \end{array}$$

### 2.3. Comparison to Summary Measures of Glycemic Variability

Second, we contrasted the semiparametric mixed model approach with commonly used summary glycemic variability measures, SD, COV, and MAGE [12]. Each measure was computed for each participant and trimester and then compared with the acceleration (and deceleration) in glucose fluctuations estimated from the semiparametric mixed model. First, SD around a mean glucose level for each of the three trimesters was computed. Next, COV was calculated by dividing the SD by the mean of the corresponding glucose readings. MAGE was computed to measure the average heights of glucose excursions between peaks (highs) and nadirs (lows) that exceed the SD for an individual within a given day. It generates a value for the variation around a mean glucose value by summing the absolute rises or falls encountered. These measures were compared between LGA and AGA groups for each trimester using repeated measure analysis of variance, in order to contrast these longitudinal findings, which are typically used to make conclusions about glycemic fluctuations, with results from the semiparametric mixed modeling.

### 2.4. Identifying Fluctuations Associated with LGA Births

Lastly, we graphically examined results from the semiparametric mixed model for the fitted curves representing LGA and AGA groups as follows. To reflect uncertainty in the estimated rates of change in glucose over pregnancy, 95% pointwise confidence intervals (CI) of first-order (velocity) and second-order (acceleration/deceleration) glucose curves were constructed using bootstrap sampling. The resulting 95% CI for each curve was used to examine differences between LGA and AGA groups as well as significance of changes in velocity and acceleration/deceleration. These results are subsequently shown as figures.

## 3. Results

**Figure 1:**
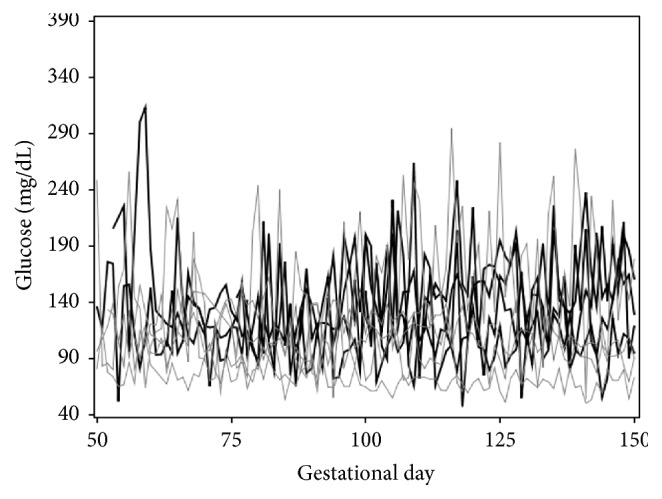
The glucose readings (*y*-axis, in mg/dL) of 5 randomly selected women with type 1 diabetes are plotted against gestation time (*x*-axis, in days). These profiles show large variation both between and within subjects during pregnancy.

The analysis cohort was comprised of a total of 199 women with type 1 diabetes with 246 pregnancies and 139,991 glucose readings. Glucose concentration recording times and number sampled varied across women, with each woman having a mean (range) number of recordings of 634 (10–2728). A random sample of five women showed large within-subject and between-subject variations in glucose levels over the gestational period (Figure 1). Duration of follow-up among women was 155 (62–182) days, between 7 and 36 weeks' gestation. Forty-three percent of the entire cohort had LGA infants. Maternal age at pregnancy was similar between LGA and AGA groups (26.5 (16–37) versus 27.0 (15–38) years).

**Figure 2:**
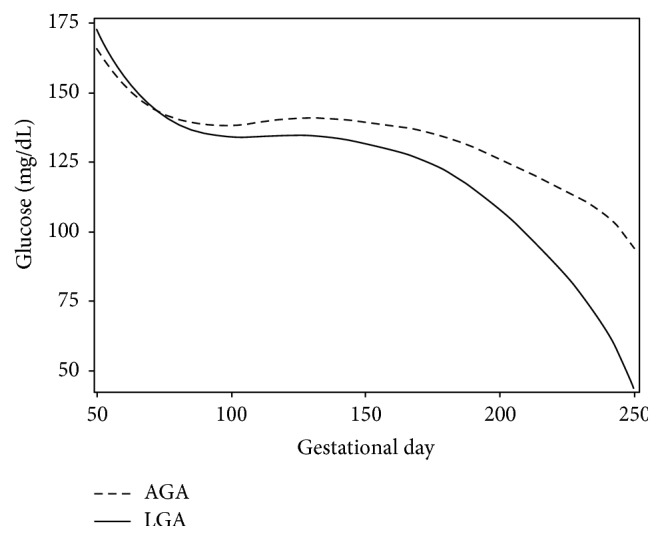
Fitted glucose curves of women with type 1 diabetes based on semiparametric regression modeling using penalized regression splines to estimate the rate of change in glucose (*y*-axis, mg/dL) against gestation time (*x*-axis, in days). The solid line represents fitted maternal glycemic profiles of mothers who gave birth to LGA infants; the dashed line represents fitted maternal glycemic profiles of mothers who gave birth to AGA infants.

For the semiparametric mixed model fitting, a total of 8 knots were selected, ranging from 76.67 to 247.99 gestational days, using the quantile method described by Ngo and Wand (2004). The fitted maternal glycemic profiles reflected differences in glucose changing across gestational days for LGA and AGA births (Figure 2). The COV, SD, and MAGE estimates by each trimester and group are presented in Table 1. For all three measures, there was a statistically significant decline in glycemic variability stratified by LGA and AGA groups over trimesters. The comparisons between LGA and AGA groups for each trimester did not yield statistically significant differences for any variability measures suggesting similar glucose fluctuations between the groups. Thus, it may be that the results from the standard methods are misleading or yield insufficient information, as the findings do not differentiate rates of progression in glucose intake for women delivering LGA versus AGA infant.

**Figure 3:**
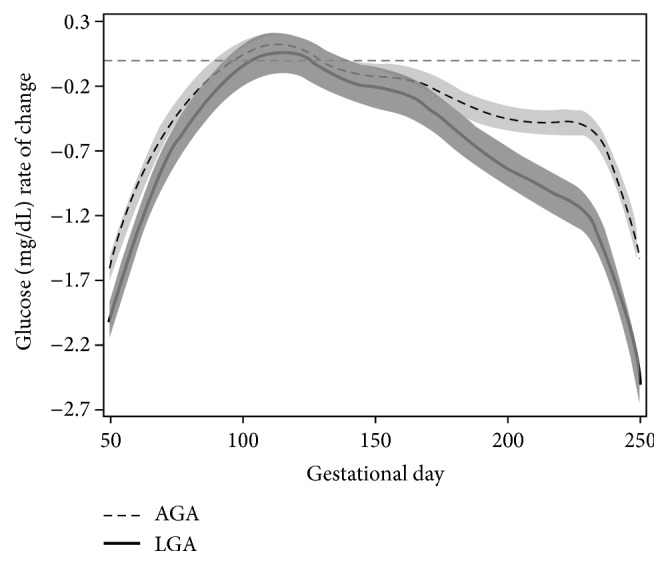
Glucose velocities for women with type 1 diabetes according to LGA (solid line) and AGA (dashed line) birth outcomes. Rates of change in glucose for each group were calculated based on the first derivative (*y*-axis, mg/dL/day) against gestation time (*x*-axis, in days). The overlapping region in 95% pointwise confidence interval indicates the gestational time period in which both LGA and AGA have similar rates of change in glucose readings. It is illustrated here that women delivering LGA infants have more rapid decline in glucose velocity.

The mean glucose concentration for both groups declined, with more attenuated decreases in the LGA group. Across gestational time, mothers who had AGA babies had smaller (i.e., less negative) rates of change or velocity in glucose level, compared to those who had LGA births (Figure 3). Greater velocity in glucose recordings for both AGA and LGA occurred between 50 and 120 gestational days (AGA: −1.62 mg/dL/day to 0.09 mg/dL/day; LGA: −2.02 mg/dL/day to 0.03 mg/dL/day). The rate of change was acute between 150 and 250 gestational days, with a more rapid decline in glucose levels for LGA births (−0.21 mg/dL/day to −2.50 mg/dL/day), compared to AGA births (−0.002 mg/dL/day to −1.54 mg/dL/day). The most rapid rate of change in glucose levels occurred between 230 and 250 gestational days for both AGA and LGA with a more rapid decline for the LGA births (AGA: −0.51 to −1.54 mg/dL/day; LGA: −1.20 to −2.50 mg/dL/day). The pointwise 95% confidence band for rate of change (or first-order derivative) of glucose readings reflected nonoverlapping regions between LGA and AGA groups for the first trimester, suggesting that the velocity of glucose readings was different for AGA and LGA births (Figure 3). However, for the second trimester, the confidence bands overlapped, reflecting the rates of change in glycemic levels were similar for LGA and AGA groups. During the third trimester the confidence bands did not overlap, meaning that the glycemic levels velocity was different, with an increasing rate of change in glucose reading for the LGA compared to the AGA group.

**Figure 4:**
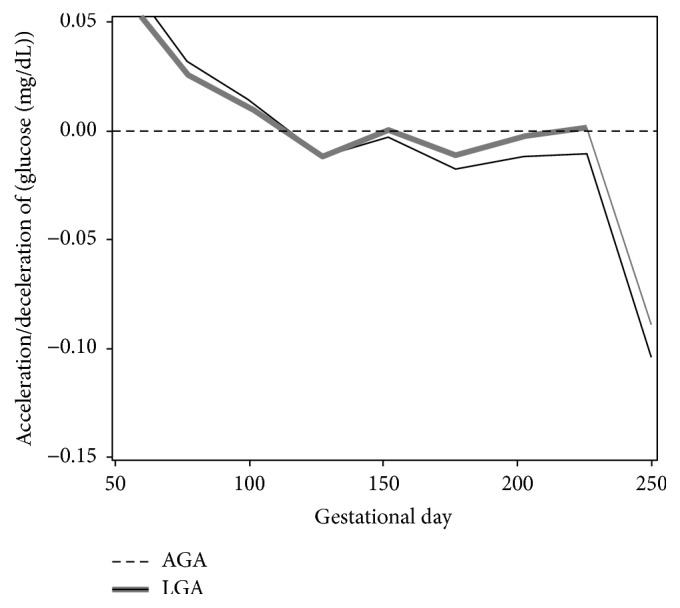
Patterns of acceleration and deceleration in glucose according to LGA (solid line) and AGA (dashed line) birth outcomes. Rates of change in glucose for each group were calculated based on the second derivative (*y*-axis, mg/dL/day^2^) against gestation time (*x*-axis, in days). Portions of each curve that are above zero imply acceleration, while portions of each curve that are below zero mark deceleration. The overlapping regions in the 95% pointwise confidence intervals indicate periods in which glycemic levels for both groups had similar acceleration or deceleration. (It should be noted that women delivering LGA infants had increased acceleration early in pregnancy and increased deceleration later in pregnancy compared with women delivering AGA infants.)

The extents of rapid changes in glucose, as measured by acceleration and deceleration, were similar initially for both LGA and AGA groups (Figure 4). Glucose curves in both groups tended to accelerate (i.e., glucose velocity was increasing) for the first 150 days of gestation. Glucose levels decelerated in both groups roughly for the rest of gestation. The deceleration in glucose during the third trimester was more attenuated for women with LGA compared to AGA births, suggesting higher risk of hypoglycemia for the former group. The narrow confidence bands for rate of change and acceleration were due to relatively large number of observations in our study.

## 4. Discussion

Development of the fetal pancreas occurs during the end of the first trimester and beginning of the second, with insulin production evident by midpregnancy [21]. It has been demonstrated in the sheep model that maternal glucose fluctuations induce fetal insulin secretion. Insulin clamp studies showed that bursts of hyperglycemia, rather than constantly maintained levels of hyperglycemia, induced significant increases in fetal arterial plasma insulin levels [22]. This shows the effect of maternal glucose transported across the placenta emphasizing the importance of fluctuations in maternal glucose levels, which we have termed as velocity and acceleration/deceleration, on fetal insulin secretion. In addition, this excess of fetal insulin secretion can, in turn, lead to excessive fetal growth and delivery of LGA infant [23].

Using a novel statistical approach, we have demonstrated that time-specific fluctuations in velocity and acceleration/deceleration (or change in velocity) of glucose levels differed across gestational age and between women delivering LGA and AGA infants. These findings were not indicated in the conventional summary measures of glucose variability. There was a steeper rate of change (or velocity) in glucose concentration between 150 and 250 gestational days for LGA group compared to AGA group. The measurement of acceleration by the second-order derivative with respect to gestation time provided instantaneous measures of glucose fluctuations over the entire pregnancy period. The acceleration in glucose levels displayed sharp decline in the first and third trimester for both LGA and AGA groups. During the first trimester, the acceleration on glucose levels was lower for AGA compared to LGA group, suggesting a potential risk of developing hypoglycemia for the former. The rebounds from hypoglycemia occurred for both groups in the second trimester, with a lower rate for LGA group. After that, there was a decline in glucose concentration for both groups, following a steady state and a sharp trough in glucose levels for LGA, suggesting risk of developing hypoglycemia during the third trimester.

Several studies have demonstrated that having glycemic fluctuations is a deterministic factor for hyperglycemic and hypoglycemic excursions. In recent years continuous glucose monitoring systems have emerged as an effective technology with an ability to monitor glucose trends over time. Involving large amount of data, continuous glucose monitoring systems provide information about frequency of fluctuations about blood glucose levels [24]. However, the unbalanced nature of blood glucose data results in large magnitude of within- and between-patient variabilities. The semiparametric regression approach in our study improved our understanding of velocity and acceleration of maternal glycemic profiles on pregnancy outcomes. The estimation of derivatives provided more accurate information on periods in which glucose has higher variation, compared to use of summary measures. An interesting feature of acceleration/deceleration in glucose levels was detecting gestation-specific recurrence of risk of developing hypoglycemia associated with mother's glycemic profiles.

Intervening when data indicate that glucose levels are outside of the target area of normal ranges to control the glycemic fluctuations delays several complications associated with diabetes. It is possible that attenuated changes in glucose are a reflection of insulin delivery. Future analysis is needed to understand the potential role of this therapy in glucose curtailment. The acceleration, which measures the variability in glucose concentration, may provide a timely warning of severe hyperglycemia or hypoglycemia. The estimates could serve as a prognostic aid to monitor glucose across the gestational period, thus increasing patients' and clinicians' abilities to take timely actions in a more accurate manner and hence improving pregnancy outcomes. The velocity and acceleration in glucose fluctuations may guide the clinicians to detect the time of intervention for continuous glucose monitoring. By precisely identifying specific time points with continuous modeling, the first- and second-order derivatives of glucose fluctuations will yield additional information that might contribute to developing risk model at different stages of pregnancy. Our approach showed that, at the early stage of pregnancy, glucose monitoring will be useful for both LGA and AGA and that at the end stage of pregnancy it is very important to monitor the glucose levels, especially to minimize the risk of LGA birth. Such information was not evident from any of the traditional glucose variability indices.

There are some limitations to the current study. Although the original protocol directed multiple daily measurements of glucose, it is difficult for a subject to check glucose at the precise moment of a hypo- or hyperglycemic excursion. Rapid changes in blood glucose levels may not be reflected in these measurements, which often occur at the end of an excursion. Thus, the measurements may not always reflect the precise level of fluctuations and may show lower readings instead. In that case, the rate of change and acceleration (or deceleration) estimates may involve underestimation of change in glucose fluctuations. This study did not include covariate adjustment. Future work should aim to estimate the effects of fixed and time-varying predictors on the glucose readings to explain the causal inference and improve our understanding of fluctuation in maternal glucose profile on pregnancy outcome. Also, use of multilevel models to analyze cases of multiple pregnancies from the same women could be investigated using semiparametric regression modeling.

## 5. Conclusions

Our study is consistent with fetal sheep clamp studies demonstrating the role of maternal glucose variability during fetal development. We demonstrate the increased sensitivity of the more sophisticated statistical approach to analyzing multiple glucose measurements, which can also be applied to information from continuous glucose monitoring. We showed that first- and second-order derivatives of glycemic levels with respect to gestational time, termed as velocity and acceleration in glucose readings, are useful to explain variability in maternal glycemic profiles with LGA and AGA births and detect gestational periods when they are different. We were able to identify specific gestational periods where rate of progression in maternal glucose concentration differed between LGA and AGA groups. The time-specific detection of velocity and acceleration (or deceleration) in glucose levels can be effective in monitoring risk for hyperglycemic (or hypoglycemic) events. Responding in a timely manner to high- and low-glucose alerts, the clinicians can significantly reduce glucose fluctuations and keep the glucose readings within target range, thereby improving pregnancy outcome.

## Supplementary Material

The Supplementary Material contains relevant SAS code for the methodology described in the article.

## Figures and Tables

**Table 1:** Measure of glucose variabilities compared between LGA and AGA babies across trimesters^*∗*^.

Measures of glucose variability	AGA EST (SE)	LGA EST (SE)
Standard deviation		
Trimester 1	80.13 (2.46)	74.64 (3.12)
Trimester 2	75.93 (2.29)	70.25 (3.00)
Trimester 3	67.19 (2.29)	62.72 (2.97)
Coefficient of variation		
Trimester 1	55.66 (1.25)	52.36 (1.50)
Trimester 2	52.49 (1.14)	48.35 (1.39)
Trimester 3	49.72 (1.13)	44.14 (1.36)
MAGE		
Trimester 1	175.38 (6.70)	166.89 (9.15)
Trimester 2	165.93 (6.45)	162.65 (8.94)
Trimester 3	158.73 (6.50)	155.31 (8.97)

*Note*. EST: estimate, SE: standard error; MAGE: mean amplitude of glycemic excursion, excursions > 1 SD from the mean. ^*∗*^AGA and LGA comparisons with respect to variability measures were not statistically significant at 0.05.
