# Supplementary material for: Glycemic Excursions in Type 1 Diabetes in Pregnancy: A Semiparametric Statistical Approach to Identify Sensitive Time Points during Gestation
Source: J Diabetes Res. 2017 Feb 8;2017:2852913. doi: 10.1155/2017/2852913 (PMC5320381; doi:10.1155/2017/2852913)
Supplement: Supplementary file 1 — The Supplementary Material contains relevant SAS code for the methodology described in the article. [file 2852913.f1.docx]

**Glycemic excursions in type 1 diabetes in pregnancy: A semiparametric statistical approach to identify sensitive timepoints during gestation**

**Online Supplement**

Resmi Gupta, MS, MA, Jane Khoury, PhD, Mekibib Altaye, PhD, Rhonda D. Szczesniak, PhD

**Appendix A. SAS implementation**

**Table A1.**

| **Variable** | **Description** |
| --- | --- |
| Glucose  Cincatwt  Id  Gestday  l1-l8 | Response variable : Glucose reading  Group variable : LGA and AGA  Subject identification number  Gestational day (time)  Columns of Z matrix (cubic) for smoothing (K=8 knots) |

A total of 8 knots were selected using the quantile algorithm from Ngo and Wand (2004), ranging from 76.67 to 247.99 gestational time.

**PROC** **HPMIXED** DATA=finaldata ;

CLASS id cincatwt;

MODEL glucose = gestdaytime gestdaytime*gestdaytime

gestdaytime*gestdaytime*gestdaytime cincatwt

cincatwt*gestdaytime

cincatwt*gestdaytime*gestdaytime

cincatwt*gestdaytime*gestdaytime*gestdaytime/s;

random intercept gestdaytime/subject=id type=cs;

random l1-l8 / type=toep(**1**) s ;

Title 'Semiparametric mixed regression model with cubic polynomial basis';

**run** ;
